# Results of 189 wrist replacements

**DOI:** 10.3109/17453674.2011.652894

**Published:** 2012-02-08

**Authors:** Astor Reigstad, Jan Mjørud

**Affiliations:** ^1^Retired Consultant, Hand and Microsurgery Section, Orthopaedic Department, Rikshospitalet, Oslo, Norway; ^2^Department of Orthopaedics, Diakonhjemmet Hospital, Oslo, Norway

*Sir*—The article “Results of 189 wrist replacements. A report from the Norwegian Arthroplasty Register” by Krukhaug at al. (2011) seems to represent a new trend in the reports of register studies. The advantage of arthroplasty register studies is the comparison of a very high number of implants operated in several clinics and thus, false results from inferior surgical skill and other nonprosthetic factors may be minimized. In the register study by Krukhaug et al. a comparison of three different wrist prostheses with 23, 76, and 90 (84) patients in each group has been carried out (the smallest group includes three different types of a developing prosthesis). This number is far beyond the lowest number of implants reported by any arthroplasty register in the literature. Only 52% of the wrist replacements performed in Norway in the same period was reported to the Arthroplasty Register compared to 99% for knee replacements according to the Norwegian Patient Register.

This underreporting of wrist replacements was published by the register already in 2006 (Espehaug et al. 2006). Therefore, it should have been possible for the register to find out what the reason for the discrepancy could be. The authors explain the reason for the underreporting to be that the Norwegian Arthroplasty Register is not so well established among wrist surgeons, but they fail to discuss what importance underreporting has for the results. According to our opinion the low number of patients and the incompleteness of data make the results in the article unreliable. We also mean that studies on so small patient groups as in this article should be carried out in clinical investigations, eventually in cooperation with the arthroplasty register.

Compared to the hip the wrist is a complicated joint, and the results after prosthetic replacement is supposed to be inferior. On the other hand the salvage procedure in the wrist is an arthrodesis (Carlson and Simmons 1998), which is not possible in the hip after a failed prosthesis. Krukhaug el al. found a prosthetic survival of 80%, and they conclude that there is no support for widespread implementation of the procedure. Without any documentation in the article, they also claim that the function with prosthesis is not substantially better than with an arthrodesis. We do not agree in this opinion, and we cannot see that the article documents anything about the wrist function after neither a prosthesis nor an arthrodesis. Much higher quality-adjusted life-years are estimated for the patients with an arthroplasty compared to arthrodesis (Cavaliere et al. 2009). Higher quality-adjusted life-years were even estimated in the theoretical extreme situation with all major complications occurring in the arthroplasty patients and no major complications occurring in fused patients. Some patients with a fused wrist have appreciable difficulties with activities of daily living (Sauerbier et al. 2000). Most of the patients with a ruined wrist will prefer mobility in the wrist with a prosthesis when the alternative is an arthrodesis although they know that a failed prosthesis will eventually led to a stiff wrist (Adey et al. 2005).

Wrist prostheses have so far been restricted to low demand rheumatoid patients. If the results of this register study are sustainable, Krukhaug et al. are the first authors to document that the outcome of wrist prosthesis in a high demand non rheumatoid group of patients is similar to that of the rheumatoid group. They offer no comment on this finding in the discussion, and there is no explanation why the non rheumatoid patients in the Biax group were excluded from the study. The only increased revision risk the authors found (female), is not discussed although this is different from prosthetic replacements in other joints.

Two clinics are responsible for 75% of the reported wrist replacements in the Norwegian Arthroplasty Register. As Krukhaug et al. know, both clinics have their own on-going follow-up studies on these patients. Cooperation with clinics which have experience with wrist replacement and wrist arthrodesis would have improved the quality of the article.
