# Supplementary material for: Results of 189 wrist replacements
Source: Acta Orthop. 2012 Feb 8;83(1):101–2. doi: 10.3109/17453674.2011.652894 (PMC3278666; doi:10.3109/17453674.2011.652894)
Supplement: Related texts

# Letter: Results of 189 wrist replacements

**Published:** 2012-02-08

**Authors:** Yngvar Krukhaug, Stein A Lie, Leif I Havelin, Ove N Furnes, Leiv M Hove

**Affiliations:** ^1^The Norwegian Arthroplasty Register, Department of Orthopaedic Surgery, Haukeland University Hospital; ^2^Section for Orthopaedic Surgery, Department of Surgical Sciences, University of Bergen; ^3^Department of Health, University Research Bergen, Bergen, Norway

*Sir*—We thank Astor Reigstad, who we understand is the inventor of the Elos and the Gibbon wrist prostheses, and Jan Mjørud for their interest in our article. We excuse the small numbers of wrist prostheses in the article (Krukhaug et al. 2011). The study included all wrist prostheses reported to the register since 1994 and it is probably the largest study on wrist prostheses in the literature. In 2009 the register received a letter from one of the users of the Elos and Gibbon prostheses. The letter was a response to an oral presentation of the study at the annual meeting at the Norwegian Orthopaedic Society, and the matter of publication of this study was therefore brought to the Board of the Norwegian Arthroplasty Register. The Board decided in 2010 to publish the article in spite of the low numbers of implants.

In your letter you argue that only 52% of the wrist replacements performed in Norway has been reported to the Arthroplasty Register. The 52% completeness was found when we compared our numbers with the Norwegian Patient Register (NPR) where the NOMESCO 2006 coding system has been used. As explained more properly in the article, the NOMESCO coding system cannot specify if the prosthesis has been inserted in the radio-carpal joint or in other joints in the carpus or hand. Thus, the NPR data most probably includes many other implants than in the radio-carpal joint.

We are of course aware of the problems of underreporting. Especially if there is more underreporting of revisions of certain prostheses brands, the results might be unreliable. But we believe that it is unlikely that underreporting could explain that the results generally were inferior to results of hip and knee prostheses.

We agree with your view that a wrist with a well functioning prosthesis probably is better than a fused wrist. But the literature is scarce, and the long term results for wrist prostheses are unknown. And we have found no studies comparing secondary wrist fusion after failed prostheses, with the results of primary wrist fusion.

We do know that the majority of wrist prostheses in our study were operated by the most experienced hand surgeons in Norway, and based on that fact and on the results in general, we do not believe that wrist prostheses should be taken into wide-spread use by the average orthopedic surgeon.

In register studies, it is a flaw that the principal and often only outcome is prosthesis survival, and we are looking forward to results from the studies on wrist prostheses in your clinics. To get a better impression of state of the art in surgery on damaged wrist joints we believe that it would be most interesting to see quality of life measures, pain and functional results, and reoperations rates in patients with wrist prostheses compared to primary wrist fusion. It is also important to compare secondary wrist fusion after failed wrist prosthesis and primary wrist fusion.

## References

[CIT0001] Adey L, Ring D, Jupiter JB (2005). Health status after total wrist arthrodesis for posttraumatic arthritis. J Hand Surg Am.

[CIT0002] Carlson JR, Simmons BP (1998). Wrist arthrodesis after failed wrist implant arthroplasty. J Hand Surg Am.

[CIT0003] Cavaliere CM, Oppenheimer AJ, Chung KC (2009). Reconstructing the rheumatoid wrist: A utility analysis comparing total wrist fusion and total wrist arthroplasty from the perspectives of pheumatologists and hand surgeons. Hand (N Y).

[CIT0004] Espehaug B, Furnes O, Havelin LI, Engesaeter LB, Vollset SE, Kindseth O (2006). Registration completeness in the Norwegian Arthroplasty Register. Acta Orthop.

[CIT0005] Krukhaug Y, Lie SA, Havelin LI, Furnes O, Hove LM (2011). Results of 189 wrist replacements. Acta Orthop.

[CIT0006] NAR (2010). The Norwegian Arthroplasty Register. http://nrlwebihelsenet/eng/defaulthtm.

[CIT0007] Sauerbier M, Kluge S, Bickert B, Germann G (2000). Subjective and objective outcomes after total wrist arthrodesis in patients with radiocarpal arthrosis or Kienbock's disease. Chir Main.
